# Effectiveness and safety of adjunctive cenobamate in people with focal‐onset epilepsy: Interim results after 24‐week observational period from the BLESS study

**DOI:** 10.1111/epi.18357

**Published:** 2025-03-15

**Authors:** Simona Lattanzi, Fedele Dono, Giuseppe d'Orsi, Alfredo D'Aniello, Mariangela Panebianco, Paolo Bonanni, Carlo Di Bonaventura, Elisa Montalenti, Antonio Gambardella, Federica Ranzato, Giada Pauletto, Elena Tartara, Angela La Neve, Francesca Bisulli, Giampaolo Vatti, Patrizia Pulitano, Claudio Liguori, Giovanni Assenza, Alfonso Giordano, Pietro Pignatta, Vincenzo Belcastro, Michela Cecconi, Simone Beretta, Chiara Pizzanelli, Marianna Pezzella, Massimo Gangitano, Maurizio Elia, Rosaria Renna, Catello Vollono, Angelo Pascarella, Luciana Tramacere, Giovanni De Maria, Daniela Audenino, Maria Pia Pasolini, Loretta Giuliano, Rosita Galli, Gionata Strigaro, Monica Puligheddu, Angelo Labate, Pietro Penza, Stefano Quadri, David Stokelj, Giovanni Boero, Elisa Fallica, Monica Santo Sabato, Giovanni Falcicchio, Nicoletta Foschi, Michela Procaccini, Valentina Villano, Gabriele Camattari, Fabiano Mele, Barbara Roncari, Giancarlo Di Gennaro, BLESS Study Group

**Affiliations:** ^1^ Department of Experimental and Clinical Medicine, Neurological Clinic Marche Polytechnic University Ancona Italy; ^2^ CAST, Center for Advanced Studies and Technology ITAB, Institute for Advanced Biomedical Technologies, Department of Neuroscience, Imaging, and Clinical Science University “G. d'Annunzio” Chieti‐Pescara Chieti Italy; ^3^ IRCCS Ospedale Casa Sollievo della Sofferenza‐UOC Neurologia San Giovanni Rotondo (FG) Italy; ^4^ IRCCS Neuromed Pozzilli (IS) Italy; ^5^ ARNAS Garibaldi‐UOC Neurologia Catania Italy; ^6^ IRCCS E. Medea Scientific Institute, Epilepsy Unit Conegliano, Treviso Italy; ^7^ Policlinico Umberto I Rome Italy; ^8^ Città della Salute e Scienza PO Molinette Turin Italy; ^9^ Azienda Ospedaliero Universitaria “Renato Dulbecco” Catanzaro Italy; ^10^ Ospedale San Bortolo Vicenza Italy; ^11^ Azienda Sanitaria Universitaria Friuli Centrale Udine Italy; ^12^ IRCCS Fondazione Mondino, Full Member ERN‐EpiCARE Pavia Italy; ^13^ Azienda Ospedaliero Universitaria Consorziale Policlinico Bari Italy; ^14^ IRCCS Istituto delle Scienze Neurologiche di Bologna, Full Member ERN‐EpiCARE Bologna Italy; ^15^ Department of Biomedical and NeuroMotor Sciences (DIBINEM) University of Bologna Bologna Italy; ^16^ Azienda Ospedaliero Universitaria Senese Policlinico Scotte Siena Italy; ^17^ AOU Policlinico Umberto I – UOC Neurofisiopatologia e Malattie Neuromuscolari, Policlinico Umberto I Rome Italy; ^18^ Neurology Unit University Hospital of Rome Tor Vergata Rome Italy; ^19^ Department of Systems Medicine University of Rome Tor Vergata Rome Italy; ^20^ Fondazione Policlinico Campus Bio‐Medico Rome Italy; ^21^ Azienda Ospedaliero Universitaria Luigi Vanvitelli Naples Italy; ^22^ Humanitas Gradenigo Turin Italy; ^23^ Ospedale Maggiore Lodi Italy; ^24^ Ospedale Santa Maria della Misericordia Perugia Italy; ^25^ Fondazione IRCCS San Gerardo dei Tintori University of Milano‐Bicocca Monza Italy; ^26^ Neurology Unit, Department of Clinical and Experimental Medicine University of Pisa Pisa Italy; ^27^ Ospedale Antonio Cardarelli Naples Italy; ^28^ Policlinico Giaccone Palermo Italy; ^29^ IRCCS Associazione Oasi Maria SS Troina (EN) Italy; ^30^ Azienda Ospedaliera di Rilievo Nazionale “A. Cardarelli” Naples Italy; ^31^ Fondazione Policlinico Universitario Agostino Gemelli, IRCCS Rome Italy; ^32^ Department of Medical and Surgical Sciences Magna Graecia University of Catanzaro Reggio Calabria Italy; ^33^ Ospedale San Giovanni di Dio Florence Italy; ^34^ Fondazione Poliambulanza Brescia Italy; ^35^ S.S.C Neurofisiopatologia E.O Ospedali Galliera Genova Genoa Italy; ^36^ ASST Spedali Civili Brescia Italy; ^37^ UOC Clinica Neurologica AOU Policlinico G. Rodolico San Marco Catania Italy; ^38^ Ospedale San Donato Arezzo Italy; ^39^ Azienda Ospedaliero Universitaria Maggiore della Carità Novara Italy; ^40^ Azienda Ospedaliero‐Universitaria Cagliari e Università di Cagliari Monserrato (CA) Italy; ^41^ Neurophysiopathology and Movement Disorders Clinic University of Messina Messina Italy; ^42^ Regional Epilepsy Center University of Messina Messina Italy; ^43^ Ambulatorio Epilessia, AOU San Giovanni di Dio e Ruggi d'Aragona Clinica Neurologica Salerno Italy; ^44^ ASST Papa Giovanni XXIII Bergamo Italy; ^45^ Azienda Sanitaria Universitaria Giuliano Isontina (ASUGI) Trieste Italy; ^46^ Ospedale SS. Annunziata Taranto Italy; ^47^ Azienda Ospedaliero Universitaria Ferrara Cona (FE) Italy; ^48^ Ospedale Vito Fazzi Lecce Italy; ^49^ Neurologia Universitaria “Puca‐Amaducci”, Azienda Ospedaliero Universitaria Consorziale Policlinico Bari Italy; ^50^ Azienda Ospedaliero Universitaria delle Marche Ancona Italy; ^51^ Angelini Pharma S.p.A Rome Italy; ^52^ IQVIA Solutions Italy SRL Modena Italy

**Keywords:** cenobamate, early use, epilepsy management, real‐world evidence, seizure reduction

## Abstract

**Objective:**

Cenobamate is an antiseizure medication (ASM) with a dual mechanism of action that was recently approved for the treatment of focal seizures in adults. This analysis aimed to describe the outcomes at 12 and 24 weeks after starting cenobamate therapy in a real‐world setting.

**Methods:**

BLESS [NCT05859854] is an ongoing, observational, retrospective and prospective cohort study to evaluate the real‐world effectiveness and safety of adjunctive cenobamate in adults with uncontrolled focal epilepsy. Subgroup analysis was performed in subjects with 2 to 3 previous ASMs (early users) and those with >3 previous ASMs (late users).

**Results:**

The second interim analysis of the BLESS study included 388 participants with a median (interquartile range) age of 43.0 (31.0–54.0) years. They had a median of 6.0 (4.0–9.0) prior ASMs and a median of 7.2 (3.0–20.6) monthly seizures at baseline. The median monthly seizure frequency was reduced by 59.9% (19.2%–87.3%) from baseline to 24 weeks; 229 (59.0%) subjects had a ≥50% seizure frequency reduction, and 44 (11.3%) showed sustained seizure freedom. The proportion of participants taking ≤2 concomitant ASMs increased from 217 (56.5%) at baseline to 239 (65.7%) at 24 weeks. Among the early users (*n* = 76, 19.6%), the median reduction in monthly seizure frequency at 24 weeks was 78.0% (50.0–97.1%), and 76.3% of subjects had a ≥50% response rate. The frequency of adverse drug reactions (ADRs) was 5.3% and 23.4% in early and late users. The most frequent ADRs were somnolence, dizziness, and balance disorder; after the occurrence of ADRs, 63.5% of participants maintained the prescribed dose, and 5.2% permanently discontinued treatment.

**Significance:**

Cenobamate was effective in reducing seizure frequency in a real‐world setting and showed a manageable safety profile. The treatment with cenobamate also reduced the burden of concomitant ASMs in both early and late users.

**Key points**

- The second interim analysis of the BLESS study included 388 participants from 46 Italian sites.
- The reduction in median monthly seizure frequency was 59.9%, and 59.0% of the participants had a ≥50% seizure frequency reduction at 24 weeks.
- The reduction in seizure frequency was higher among participants with 2 to 3 previous antiseizure medications (early users).
- Seizure freedom was sustained for 24 weeks by 22.4% of early users and 8.7% of participants with >3 previous antiseizure medications.
- A reduction in the number of concomitant antiseizure medications was observed over 24 weeks of treatment with cenobamate.

## 1 INTRODUCTION

Epilepsy is one of the most prevalent neurological disorders worldwide.^1^ Despite the variety of antiseizure medications (ASMs) and non‐pharmacological treatments available, ~30% of people with epilepsy (PwE) continue to experience seizures.^2^, ^3^ Of note, persistent seizures impact health‐related quality of life (HRQoL) and burden the social and life wellness of PwE.^4^

ASMs that reduce seizure occurrence while maintaining good tolerability can favorably change the QoL of PwE and enhance their everyday life. Since its approval in Europe, a substantial body of evidence has been collected for regarding the efficacy and safety of cenobamate in the treatment of focal‐onset seizures in PwE whose seizures have not been adequately controlled despite a history of treatment with at least two ASMs. Data from the real‐world evidence^5^, ^6^, ^7^, ^8^, ^9^ were overall consistent with data from clinical trials.^10^, ^11^, ^12^, ^13^, ^14^ In most current real‐world studies, the average number of ASMs administered before starting cenobamate was high.^7^, ^15^ These data confirm efficacy in PwE at a late stage of the disease, but more real‐world evidence of the usefulness of cenobamate at an early stage is now accruing.^16^ Given the recent market authorization of cenobamate and the still‐limited real‐world experience, it is crucial to gather additional evidence on its effectiveness and safety when used in clinical practice at different stages over the epilepsy course.

The “Cenobamate in Adults with Focal‐Onset Seizures” (BLESS) study [ClinicalTrials.gov Identifier: NCT05859854] is an ongoing, multicenter, observational cohort study to evaluate the effectiveness, tolerability, and safety of adjunctive cenobamate in adults with uncontrolled focal epilepsy over a 52‐week period in the context of clinical practice. The study planned to involve 50 Italian sites and recruit 1200 participants.^17^ The first interim analysis included 40 participants enrolled until June 2023 and focused on 12‐week outcomes.^17^

In this article, we presented the results of the second interim analysis of the BLESS study. This analysis aimed to evaluate the outcomes at two distinct assessment time points, namely 12 and 24 weeks after the start of cenobamate therapy. In addition, a stratified analysis according to the previous treatment history included the subgroups of participants with 2 to 3 previous ASMs (early cenobamate users) and those with >3 previous ASMs (late users).

## 2 METHODS

### 2.1 Study design

The BLESS study is a multicenter, observational, retrospective and prospective cohort study. The study involves 50 sites specialized in the management of epilepsy in Italy, started on January 24, 2023, and is ongoing. At the time of the data extraction for this interim analysis, there were 46 recruiting sites. The study objectives, design, and eligibility criteria have been described previously.^17^ Briefly, the study included male or female adults (age ≥18 years) with uncontrolled focal epilepsy despite treatment with at least 2 ASMs before cenobamate initiation, as per Summary of Product Characteristic. At enrollment, all participants were treated for at least 12 weeks with adjunctive cenobamate independently from study participation and should have completed the titration period up to the initial recommended target daily dose of 200 mg. According to the observational design, the target dose could be reached after 12 weeks, based on the clinical decision of treating physician. The index date (i.e., the date of cenobamate initiation) was at least 12 weeks but no more than 52 weeks before the enrollment.

All participants signed an informed consent form before data collection, in accordance with the Declaration of Helsinki. The ethics approval was granted by all local ethics committees (first approval of the coordinating ethics committee – Comitato Etico IRCCS Istituto Neurologico Mediterraneo Neuromed – on September 29, 2022). The study was registered at ClinicalTrials.gov [NCT05859854].

### 2.2 Data collection

Data from the index date to the enrollment date were collected retrospectively. After enrollment, the data collection was prospective for up to 52 weeks. The outcomes after the index date were recorded through electronic case report forms at five time points: week 12, week 24, week 52, week 76, and week 104 after cenobamate initiation. All the details about sample size justification, data collection, and applicable regulations were reported elsewhere.^17^

### 2.3 Study outcomes

The primary objective of the BLESS study was to determine the intra‐patient percent change and the proportion of participants with a ≥50% reduction in monthly seizure frequency from the pretreatment baseline over 52 weeks. Secondary outcomes included the proportion of participants with a ≥75%, ≥90%, or 100% reduction and sustained reduction in monthly seizure frequency from the pretreatment baseline and the safety and tolerability of cenobamate as an adjunctive treatment. The full list of secondary and explorative objectives has been reported previously.^17^

In this interim analysis, the outcomes were analyzed at baseline (index date), 12 and 24 weeks in all evaluable subjects and according to the number of previous ASMs. The following subgroups were identified: participants with 2–3 previous ASMs (early users) and participants with >3 previous ASMs (late users).

### 2.4 Statistical analysis

The BLESS study planned descriptive statistics for all evaluable subjects. Collected data are presented as frequency (percentage and count), mean and standard deviation (SD), and median and interquartile range (IQR), according to the type of variables considered.

The second interim analysis evaluated participants who were enrolled until June 6, 2024 (database extraction date) and had a calculable percentage change in baseline monthly seizure frequency at 24 weeks (hereafter called “evaluable subjects”) (Figure S1). Primary and secondary outcomes were analyzed at baseline and at 12‐week and 24‐week observation periods.

The seizure frequency during the pre‐treatment baseline period (defined as the number of seizures occurring within the 12 weeks before the index date) and post‐baseline period was calculated by summing the total number of seizures (of all types) reported within each considered period and dividing it by the number of days in that period, excluding days with no available data. To standardize the frequency to a 4‐week period (“monthly seizure frequency”), the total seizures were multiplied by 28.

Analyses were performed using SAS Enterprise Guide version 8.2 and SAS 9.4 (SAS Institute, Cary, NC, USA). Study design and conduct, data monitoring, eCRF setup, and statistical analyses were performed by IQVIA Solutions Italy SRL, on behalf of Angelini Pharma.

## 3 RESULTS

### 3.1 Overall population

#### 3.1.1 Baseline characteristics and ASMs

Among 612 subjects enrolled at 46 Italian sites, 388 were evaluable for the second interim analysis (Figure S1). The median age was 43.0 (31.0–54.0) years, and 52.3% (*n* = 203) were women. The median age at diagnosis of epilepsy was 17.5 (8.0–30.0) years and the median number of ASMs used before cenobamate was 6.0 (4.0–9.0). The median number of seizures per month before cenobamate initiation was 7.2 (3.0–20.6). Baseline characteristics are summarized in Table 1 and medical conditions at cenobamate treatment initiation are listed in Table S1.

**TABLE 1:** Baseline characteristics of study participants.

	All (*N* = 388)	2–3 previous ASMs (*N* = 76)	>3 previous ASMs (*N* = 312)
Clinical baseline characteristics			
Age at index date (years)			
Median (IQR)	43.0 (31.0–54.0)	41.0 (29.0–52.0)	43.5 (31.0–54.0)
Sex, *n* (%)			
Male	185 (47.7)	43 (56.6)	142 (45.5)
Female	203 (52.3)	33 (43.4)	170 (54.5)
Age at diagnosis of epilepsy (years)			
Median (IQR)	17.5 (8.0–30.0)	29.5 (17.5–42.5)	15.0 (8.0–25.5)
Epilepsy etiology^a^, *n* (%)			
Structural	226 (58.2)	39 (51.3)	187 (59.9)
Genetic or presumed genetic	12 (3.1)	0 (0.0)	12 (3.8)
Infection	5 (1.3)	1 (1.3)	4 (1.3)
Metabolic	2 (0.5)	1 (1.3)	1 (0.3)
Immune	5 (1.3)	1 (1.3)	4 (1.3)
Unknown^b^	149 (38.4)	34 (44.7)	115 (36.9)
Seizure type^a^, *n* (%)			
Focal aware	112 (28.9)	29 (38.2)	83 (26.6)
Focal impaired awareness	309 (79.6)	56 (73.7)	253 (81.1)
Focal to bilateral tonic–clonic	109 (28.1)	27 (35.5)	82 (26.3)
Focal, unspecified type	10 (2.6)	1 (1.3)	9 (2.9)
Motor	263 (67.8)	47 (61.8)	216 (69.2)
Non‐motor	204 (52.6)	48 (63.2)	156 (50.0)
Pre‐treatment baseline monthly seizure frequency^b^ (seizure episodes)			
Median (IQR)	7.2 (3.0–20.6)	4.3 (1.8–15.5)	8.0 (3.5–23.3)
Treatment status at baseline			
History of use of sodium‐blocking ASM, *n* (%)			
Yes	370 (95.4)	70 (92.1)	300 (96.2)
No	18 (4.6)	6 (7.9)	12 (3.8)
Number of previous ASMs used before cenobamate initiation, median (IQR)	6.0 (4.0–9‐0)	3.0 (2.0–3.0)	7.0 (5.9–9.0)
Participants per any number of previous ASMs, *n* (%)			
2 ASMs	36 (9.3)	36 (47.4)	
3 ASMs	40 (10.3)	40 (52.6)	
4 ASMs	45 (11.6)		45 (14.4)
≥5 ASMs	267 (68.8)		267 (85.6)
Previous participation in the cenobamate Italian Compassionate Use Program (CUP), *n* (%)			
No	380 (97.9)	75 (98.7)	305 (97.8)
Yes	8 (2.1)	1 (1.3)	7 (2.2)

*Note*: Current ILAE seizure and epilepsy classification schemes were used according to the standard clinical practice of participating sites. The reported results refer to the second interim analysis of BLESS study.

Abbreviations: ASM, antiseizure medication; CUP, Compassionate Use Program; ILAE, International League Against Epilepsy; IQR, interquartile range.

^a^ Subjects could have more than one condition of interest.

^b^ Epilepsy onset without relevant abnormalities on examination, cognition, history.

#### 3.1.2 Cenobamate and concomitant ASMs to cenobamate

At 24 weeks after cenobamate initiation, 79.5% (*n*/*N* = 304/383) of participants with available data were prescribed 200 mg or more of cenobamate daily. The proportion of participants who reached the recommended initial target dose (i.e., 200 mg daily of cenobamate) was 63.0% (*n*/*N* = 244/387) at 12 weeks and 67.4% (*n*/*N* = 258/383) at 24 weeks. In addition, the proportion of subjects who increased the daily dose above 200 mg remained limited (1.1%, *n*/*N* = 4/387 at 12 weeks and 14.4%, *n*/*N* = 46/383 at 24 weeks). Participants treated with >300 mg daily at 24 weeks were 0.56% (*n*/N = 2/383). Nine participants (2.3%, *n*/*N* = 9/383) permanently discontinued cenobamate between 12 and 24 weeks from the index date. The primary reasons reported for discontinuation were lack of therapeutic efficacy (*n* = 3); adverse event (AE, *n* = 2); lack of adherence (*n* = 2); seizure frequency unchanged (*n* = 1), and patient request (*n* = 1) (Table 2).

**TABLE 2:** Cenobamate and concomitant anti‐seizure medications at start of treatment and at 12 weeks and 24 weeks after treatment initiation.

	All (*N* = 388)			2–3 previous ASMs (*N* = 76)			>3 previous ASMs (*N* = 312)		
	Index date	12 weeks	24 weeks	Index date	12 weeks	24 weeks	Index date	12 weeks	24 weeks
Daily dose prescribed (mg/day) (participants per dose)^a^, *n* (%)									
<200	388 (100.0)	139 (35.9)^b^	70 (18.3)^c^	76 (100.0)	25 (32.9)^d^	12 (15.8)^e^	312 (100.0)	114 (36.7)^d^	58 (18.9)^e^
200	–	244 (63.0)	258 (67.4)	–	50 (65.8)	59 (77.6)	–	194 (62.4)	199 (64.8)
250	–	3 (0.8)	30 (7.8)	–	1 (1.3)	3 (3.9)	–	2 (0.6)	27 (8.8)
300	–	1 (0.3)	14 (3.7)	–	0 (0.0)	2 (2.6)	–	1 (0.3)	12 (3.9)
350	–	0 (0.0)	1 (0.3)	–	0 (0.0)	0 (0.0)	–	0 (0.0)	1 (0.3)
400	–	0 (0.0)	1 (0.3)	–	0 (0.0)	0 (0.0)	–	0 (0.0)	1 (0.3)
Permanent discontinuation before time point	–	0 (0.0)	9 (2.3)^f^	–	0 (0.0)	0 (0.0)	–	0 (0.0)	9 (2.9)^f^
Missing	–	1	5	–	0	0	–	1	5
Median number of ASMs concomitant to cenobamate^g^, median (IQR)	2.0 (2.0–3.0)	2.0 (2.0–3.0)	2.0 (2.0–3.0)	2.0 (1.0–2.0)	2.0 (1.0–2.0)	2.0 (1.0–2.0)	3.0 (2.0–3.0)	2.0 (2.0–3.0)	2.0 (2.0–3.0)
Overall number of ASMs concomitant to cenobamate (participants per number of ASMs)^a^, ^g^, *n* (%)									
1	61 (15.9)	73 (19.0)	86 (22.3)	28 (36.8)	28 (36.8)	33 (43.4)	33 (10.7)	45 (14.6)	53 (17.2)
2	156 (40.6)	166 (43.1)	167 (43.4)	38 (50.0)	40 (52.6)	36 (47.4)	118 (38.3)	126 (40.8)	131 (42.4)
3	120 (31.3)	112 (29.1)	102 (26.5)	10 (13.2)	8 (10.5)	7 (9.2)	110 (35.7)	104 (33.7)	95 (30.7)
4	39 (10.2)	29 (7.5)	26 (6.8)	0 (0.0)	0 (0.0)	0 (0.0)	39 (12.7)	29 (9.4)	26 (8.4)
5	6 (1.6)	3 (0.8)	3 (0.8)	0 (0.0)	0 (0.0)	0 (0.0)	6 (1.9)	3 (1.0)	3 (1.0)
6	2 (0.5)	2 (0.5)	1 (0.3)	0 (0.0)	0 (0.0)	0 (0.0)	2 (0.6)	2 (0.6)	1 (0.3)
Missing	4	3	3	0	0	0	4	3	3

*Note*: Index date: date of initiation of cenobamate treatment.

Abbreviations: ASM, anti‐seizure medication; eCRF, electronic Case Report Form; IQR, interquartile range.

^a^ Percentages were computed excluding subjects with missing data from the total.

^b^ Details of cenobamate titration dose prescribed before reaching the target dose of 200 mg daily were not collected in the eCRF in 137 subjects. Cenobamate dose was reduced to <200 mg daily at 12 weeks, after achieved this target dose during observation for *n* = 2 subjects.

^c^ In 15 subjects, cenobamate dose was reduced to <200 mg daily at 24 weeks, after achieving the target dose during observation.

^d^ Details of cenobamate titration dose prescribed prior to reaching the target dose of 200 mg daily were not collected in the eCRF in 24 subjects included in the early user subgroup (2 to 3 previous ASMs) and 113 subjects included in the late user subgroup (>3 previous ASMs).

^e^ Details of cenobamate titration dose prescribed prior to reaching the target dose of 200 mg daily were not collected in the eCRF in 11 subjects included in the early user subgroup (2 to 3 previous ASMs) and 44 subjects included in the late user subgroup (>3 previous ASMs).

^f^ Nine subjects discontinued permanently cenobamate between 12 and 24 weeks from the index date, primarily due to: lack of therapeutic efficacy (*n* = 3); adverse event (*n* = 2); lack of adherence (*n* = 2); seizure frequency unchanged (*n* = 1); patient request (*n* = 1).

^g^ Cenobamate was not counted as concomitant therapy.

At the index date and throughout the following time points, the overall median number of concomitant ASMs was stable at 2.0 (2.0–3.0). Of note, the proportion of participants who were treating with 2 or less concomitant ASMs increased from 56.5% (*n*/*N* = 217/384 subjects with available data) at the index date to 62.1% (*n*/*N* = 239/385) and 65.7% (*n*/*N* = 253/385) at 12 and 24 weeks, respectively. Lacosamide, carbamazepine, brivaracetam, and perampanel were the most frequent concomitant treatments (frequency >20%) at the different time points. Of interest, the proportion of participants treated with concomitant ASMs has decreased gradually from the index date to 24 weeks; these findings may suggest a reduction in drug load at 24 weeks (Table S2).

#### 3.1.3 Effectiveness of adjunctive cenobamate

**FIGURE 1:**
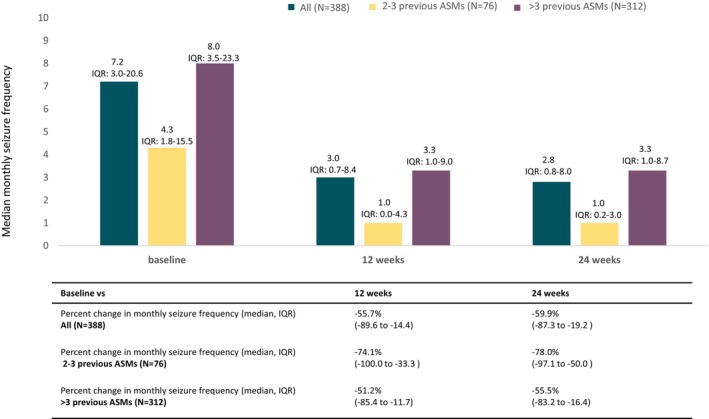
Median monthly seizure frequency at baseline and at 12 and 24 weeks from starting treatment with cenobamate in the overall study cohort, in subjects with 2 to 3 previous antiseizure medications (early users) and in subjects with >3 previous antiseizure medications (late users). ASMs, antiseizure medications; IQR, interquartile range.

Figure 1A shows the monthly seizure frequency change from pre‐treatment baseline to 12‐ and 24‐week assessment. In the overall population, the median intra‐patient percentage reduction in monthly seizure frequency was 55.7% (−89.6 to −14.4) at 12 week and 59.9% (−87.3 to −19.2) at 24 week follow‐up respect to the baseline (Figure 1A).

**FIGURE 2:**
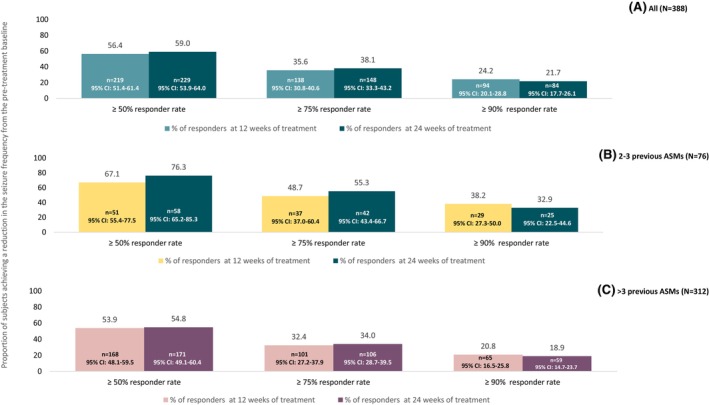
Rates of ≥50%, ≥75%, and ≥90% reduction in monthly seizure frequency from the pre‐treatment baseline in the study cohort (A), in subjects with 2 to 3 previous antiseizure medications (early users) (B), and in subjects with >3 previous antiseizure medications (late users) (C) Percentages were computed over the total number of evaluable subjects in each subgroup. ASMs, antiseizure medications; CI, confidence interval.

**FIGURE 3:**
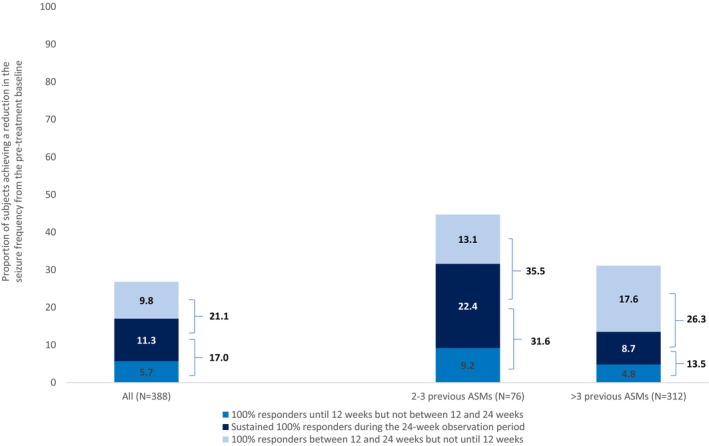
Rates of 100% reduction in monthly seizure frequency from the pre‐treatment baseline during the first 24 weeks of treatment with cenobamate. Percentages were computed over the total number of evaluable subjects in each subgroup. ASMs, antiseizure medications.

At 24 weeks, 59.0% of participants (*n* = 229) had a ≥50% response rate (Figure 2A), and 11.3% (*n* = 44) were seizure‐free without interruption through the considered observation period (sustained seizure freedom) (Figure 3). Notably, the proportion of participants with a 100% response rate was 17% (*n* = 66) during the observation period from baseline to 12 weeks, and 21.1% (*n* = 82) between 12 and 24 weeks (new responders after 12 weeks) (Figure 3).

### 3.2 Early users (2–3 previous ASMs) and late users (>3 previous ASMs)

#### 3.2.1 Baseline characteristics and ASMs

Among the 388 evaluable participants for this interim analysis, 19.6% (*n* = 76) were early users and 80.4% (*n* = 312) were late users. The early users showed a median pre‐treatment baseline monthly seizure frequency of 4.3 (1.8–15.5) episodes and a median number of previous ASMs of 3.0 (2.0–3.0), equally distributed between 2 (47.4%, *n* = 36) and 3 (52.6%, *n* = 40) previous ASMs. The late users had a median number of baseline monthly seizures of 8.0 (3.5–23.3) episodes and 7.0 (5.0–9.0) previous ASMs before cenobamate initiation (Table 1).

#### 3.2.2 Cenobamate and concomitant ASMs

At 24 weeks after cenobamate initiation, 93.4% (*n* = 71) of early users were prescribed with 200 mg of cenobamate or less. The proportion of early users who reached the recommended target dose (i.e., ≥200 mg of cenobamate) was 67.1% (*n* = 51) at 12 weeks, whereas 32.9% (*n* = 25) were treated with <200 mg of cenobamate. The proportion of early users reaching the target dose of cenobamate progressively increased, with 84.2% (*n* = 64) of participants treated with ≥200 mg of cenobamate at 24 weeks. Of note, the proportion of early users who increased the daily dose above 200 mg remained limited (1.3%, *n* = 1 and 6.5%, *n* = 5 at 12 and 24 weeks, respectively) (Table 2). A similar trend was observed among the late users: 83.7% (*n*/*N* = 257/307) of late users with available data were treated with 200 mg of cenobamate or less at 24 weeks. The proportion of late users who reached the recommended target dose (i.e., ≥200 mg of cenobamate) was 63.3% (*n*/*N* = 197/311) at 12 weeks and 78.2% (*n*/*N* = 240/307) at 24 weeks. The proportion of late users who increased the daily dose above 200 mg remained limited, with only 4.6% (*n*/*N* = 14/307) of treated with ≥300 mg. Nine participants (2.9%, *n*/*N* = 9/307) discontinued permanently cenobamate between 12 and 24 weeks from the index date (Table 2).

After cenobamate initiation, the median number of concomitant ASMs remained stable at 2.0 (1.0–2.0) from the index date throughout the subsequent time points in the subgroup of early users. Instead, the late users showed a reduction of the median number of concomitant ASMs from 3.0 (2.0–3.0) to 2.0 (2.0–3.0) at both 12‐ and 24‐week time points. It is, noteworthy, however, that the proportion of participants who withdrew at least one concomitant ASMs (i.e., took ≤2 ASMs concomitant to cenobamate during the observation period) increased in both early and late users: from 86.8% (*n* = 66) and 49.0% (*n* = 151) at baseline, to 89.4% (*n* = 68) and 55.4% (*n* = 171) at 12 weeks, and to 90.8% (*n* = 69) and 59.6% (*n* = 184) at 24 weeks, respectively. Lacosamide, carbamazepine, and levetiracetam were the most frequent concomitant treatments in early users (frequency >20%) at the different assessment time points, whereas in the late users, the most frequent concomitant treatments were lacosamide, carbamazepine, brivaracetam, and perampanel (Table S2).

#### 3.2.3 Effectiveness of adjunctive cenobamate

Figure 1B and C show the change in the median monthly seizure frequency from baseline to 12‐ and 24‐week follow‐up. In the early user subgroup, the median seizure frequency decreased to 1.0 (0.2–3.0) episode per month at 24 weeks. The median intra‐patient percentage reduction from baseline was 78.0% (50.0–97.1%) from baseline to 24‐week follow‐up (Figure 1B). In late users, the monthly seizure frequency was reduced to 3.3 (1.0–8.7) at 24 weeks, with a median intra‐patient reduction from baseline of 55.5% (16.4–83.2%) (Figure 1C).

At 24 weeks, 76.3% of early users (*n* = 58) and 54.8% of late users (95% confidence interval [CI]: 49.1–60.4) had a ≥50% reduction in baseline seizure frequency (Figure 2B,C). Sustained seizure freedom was achieved by 22.4% (*n* = 17) of early users and 8.7% (95% CI: 5.8–12.3, *n* = 27) of late users (Figure 3). In the two subgroups the proportion of participants with a 100% response rate was 9.2% (*n*/*N* = 7/76) and 4.8% (*n*/*N* = 15/312) from baseline to 12 weeks and 13.1% (*n*/*N* = 10/76) and 17.6% (*n*/*N* = 55/312) between 12 and 24 weeks (new responder after 12 weeks), respectively (Figure 3).

### 3.3 Safety of adjunctive cenobamate

During the first 24 weeks of observation, 144 AEs were recorded in 23.5% (*n* = 91) of evaluable participants. A total of 115 events were adverse drug reactions (ADRs) to cenobamate and occurred in 19.8% (*n* = 77) of participants. A total of 110 ADRs occurred in 23.4% (*n*/*N* = 73/312) of late users, and 5 ADRs occurred in 5.3% (*n*/*N* = 4/76) of early users. No serious adverse events (SAEs) were reported. Looking at the actions taken after the occurrence of ADRs, 63.5% (*n* = 73) of participants maintained the prescribed dose, and 5.2% (*n* = 6) permanently discontinued cenobamate (all late users). (Table 3) The full description of ADRs according to the Medical Dictionary for Regulatory Activities (MedDRA) Version 26.0, March 2023, is available in Table S3. The most frequent ADRs were somnolence (9.8%, *n* = 38), dizziness (2.6%, *n* = 10), and balance disorder (2.1%, *n* = 8).

**TABLE 3:** Safety events during treatment with adjunctive cenobamate until 24 weeks.

Safety events description, *n* (%)^a^	All (*N* = 388)	2–3 previousASMs (*N* = 76)	>3 previous ASMs (*N* = 312)
Participants with at least one adverse event	91 (23.5)	8 (10.5)	83 (26.6)
Participants with at least one ADR to cenobamate	77 (19.8)	4 (5.3)	73 (23.4)
Total number of adverse events	144	10	134
Total number of ADRs to cenobamate	115	5	110
Total number of serious adverse events/serious ADRs	0	0	0
Action taken with cenobamate after an ADR, *n* (%)			
Dose not changed	73 (63.5)	3 (60.0)	70 (63.6)
Dose reduced	36 (31.3)	2 (40.0)	34 (30.9)
Treatment permanently discontinued	6 (5.2)	0 (0.0)	6 (5.5)

Abbreviations: ADR, adverse drug reaction; ASM, anti‐seizure medication.

^a^ A patient could have experienced more than one ADR until 24 weeks after cenobamate treatment initiation.

## 4 DISCUSSION

The second interim analysis of the BLESS study built up the findings of the first interim analysis by evaluating a larger number of participants (388 vs 40 evaluable participants in the first interim analysis) enrolled across a greater number of Italian sites (46 vs 7 sites) with a longer length of observation.^17^

Almost 60% of the overall population showed a ≥50% reduction in monthly seizure frequency from the pre‐treatment baseline to 24 weeks. The monthly seizure frequency reduction was already significant at 12 weeks and maintained at 24 weeks, with a median percentage reduction of 59.9%. Although ~80% of participants achieved the recommended target daily dose of cenobamate (≥200 mg) at 24 weeks, the reduction in the monthly seizure frequency was already measurable. Therefore, cenobamate seems to express its antiseizure efficacy early after its introduction even before the target dose is reached. These results gain further importance considering that the evaluable subjects in this interim analysis represented a population with difficult‐to‐treat epilepsy with a median number of 6 previous ASMs and a history of 5 or more prior ASMs in almost 70% of the cases. Of interest, the results showed that new‐generation drugs like lacosamide, brivaracetam, and perampanel were widely used. This observation may be attributed to the relatively recent data collection period (from January 2023 to June 2024).

The effectiveness of cenobamate was higher in the early user subgroup, as expected: their median number of monthly seizures decreased from 4.3 to 1, and 76.3% of participants were ≥50% responders at 24 weeks. This supports the recent findings published by Winter and colleagues regarding the adoption of cenobamate in an early therapy line. In this setting, cenobamate showed the best response and seizure‐freedom rates vs other ASMs (i.e., lacosamide, levetiracetam, topiramate, and valproate).^16^ Moreover, in this real‐world study, an inverse relationship between the response to cenobamate as an add‐on therapy and the number of previous treatments was observed. This effect has been reported previously with other ASMs.^18^, ^19^

Given the importance of full seizure control to achieve a good QoL,^20^ these findings underscore the potential of cenobamate as a promising early treatment option to achieve seizure freedom and alleviate the burden of the disease, providing reassuring insights about epilepsy care.

Analyses of the overall population and the subgroups highlighted a progressive reduction in the use of concomitant ASMs from baseline to 12 and 24 weeks. The reduction in concomitant drug load was even more marked for the late user subgroup, where the proportion of participants treated with ≤2 ASMs increased up to ~60%. A growing body of evidence has demonstrated that adjunctive cenobamate therapy allows reduction of concomitant ASMs.^15^, ^21^, ^22^, ^23^, ^24^ Aboumatar and colleagues recently published a post hoc analysis of 240 subjects treated with cenobamate after a 12‐ and 24‐month observation period. In this population, the subjects reduced the number of concomitant ASMs by more than 30%, while maintaining the responder rate.^24^ In the extension of the Spanish observational study by Villanueva et al., the proportion of subjects with multiple concomitant ASMs decreased from about 62% to 14% after 12 months of treatment with cenobamate.^15^ In a German real‐world study, the number of concomitant ASMs after 1 year of treatment with cenobamate was reduced in about 40% of enrolled subjects.^23^ Similarly, in the recent analysis of data from the Italian Expanded Access Programme, up to 46.9% of subjects reduced the number of concomitant ASMs after 12 months, with a defined daily dose reduction of 22%.^9^ In the context of real‐world settings, this effect was evident also in specific subsets of PwE: Friedo et al. demonstrated cenobamate effectiveness and a concomitant reduction of overall drug load, which improved the QoL, in intellectually disabled people.^25^ It is reasonable to think that the effectiveness of cenobamate over weeks can lead to a decrease in the number of concomitant ASMs. For people with difficult‐to‐treat epilepsy, polytherapy is the usual approach. In this regard, maintaining the effectiveness with the fewest number of ASMs leads to different advantages, such as avoiding overtreatment and drug–drug interactions, reducing the impact on cognition, and improving treatment adherence.^21^, ^22^, ^23^ The reduction of concomitant ASMs was also found to minimize the occurrence of AEs and improve the retention rate. In particular, reducing the dose of concomitant ASMs early during the titration of cenobamate can be indicated in the case of phenobarbital, clobazam, and sodium‐channel blocker ASMs to limit the risk of drug–drug interactions and the development of side effects like somnolence, dizziness, and balance disorders.^26^

In this second interim analysis, the safety profile of cenobamate at 24 weeks was predictable and manageable. The most common ADRs included somnolence, dizziness, and balance disorder, which were in line with the previous evidence and with the prescribing information of cenobamate; no SAEs were observed and no cases of drug reaction with eosinophilia and systemic symptoms (DRESS) occurred. The ADRs were mild or moderate, and most of the participants who experienced an ADR continued the treatment with cenobamate at the prescribed dose; a dose reduction was needed in less than one‐third of the cases and permanent discontinuation of treatment was infrequent. Of interest, more ADRs occurred in late compared to early users; the overall higher number of concomitant ASMs may have contributed to this difference. These ADRs were in line with the previous evidence and with the prescribing information of cenobamate.

### 4.1 Strengths and limitations

The current interim analysis has some limitations that should be acknowledged. First, the BLESS study is ongoing, and the plan is to enroll 1200 subjects consecutively. So, these preliminary results, including subgroup analyses, are purely descriptive and not suitable for statistical inference. Consecutive enrollment may also help to mitigate the selection bias. Second, it is important to note that due to the observational nature of the study, seizure counts may not be always accurate. Nonetheless, to prevent underreporting, the number of monthly seizures and the assessed time period were collected in clinical charts or through participants’ diaries. The prompt data collection over a short timeframe (from January 2023 to June 2024) could depict an updated scenario on the treatment of uncontrolled focal epilepsy without applying restriction on the type or number of concomitant ASMs. Nevertheless, the national nature of the study, which included about 50 sites in Italy, can limit the generalizability of the data worldwide.

The BLESS study protocol does not plan to collect seizure frequency for each seizure type and evaluate the effectiveness of cenobamate according to seizure characteristics. The potential impact of early tolerability cannot be assessed due to the inclusion of subjects after at least 12 weeks of treatment. Similarly, as the decision‐making proceeded during the titration period, we could not identify the factors that may have contributed to the delay in reaching the target dose. Conversely, the impact of this inclusion criterion on the assessment of the effectiveness should be negligible because the discontinuation before the 12 weeks of cenobamate treatment is unlikely to be related to lack of efficacy. Data from patients who had been receiving treatment for at least 12 weeks were collected even though patients stopped treatment before enrollment.

This interim analysis also did not focus on the reasons for discontinuation of the concomitant ASMs during observation. Additional analyses looking at the reasons underlying the withdrawal of concomitant ASMs, the baseline  characteristics of participants according to their response status, and the occurrence of AEs according to different concomitant ASMs will be performed at the end of the study. Finally, changes in the total daily drug load at each time point could not be described because the study collected dose changes over time only for cenobamate.^9^ Secondary outcomes of the BLESS study, such as subjective QoL, daytime sleepiness, anxiety, and depression at the different time points will be described in future analyses.

## 5 CONCLUSION

This second interim analysis of the BLESS study described the 24‐week effectiveness of cenobamate in addition to other concomitant ASMs in adults with focal seizures treated in a real‐world setting. In participants with a history of 2 to 3 previous ASMs, cenobamate was associated with a greater seizure frequency reduction and a better tolerability compared to participants who received cenobamate after a greater number of lifetime ASMs. Furthermore, this analysis showed a reduction in the concomitant drug load already at 12 and 24 weeks after the start of the cenobamate treatment in both early and late users. No new safety signals were reported, confirming the favorable safety profile of the drug in everyday clinical practice.

## AUTHOR CONTRIBUTIONS

All persons who met authorship criteria are listed as authors, and all authors certify that they have participated in the concept, design, analysis, writing, or revision of the manuscript. All authors have approved the final version of the manuscript to be published. Michela Procaccini, Valentina Villano, Gabriele Camattari, Barbara Roncari, Fabiano Mele, Simona Lattanzi, and Giancarlo Di Gennaro conceptualized the design of the study, performed the statistical analysis, and carried out data interpretation. Fedele Dono, Giuseppe d'Orsi, Giancarlo Di Gennaro, Alfredo D'Aniello, Mariangela Panebianco, Paolo Bonanni, Carlo Di Bonaventura, Elisa Montalenti, Antonio Gambardella, Federica Ranzato, Giada Pauletto, Elena Tartara, Angela La Neve, Francesca Bisulli, Giampaolo Vatti, Patrizia Pulitano, Claudio Liguori, Giovanni Assenza, Alfonso Giordano, Pietro Pignatta, Vincenzo Belcastro, Michela Cecconi, Simone Beretta, Chiara Pizzanelli, Marianna Pezzella, Massimo Gangitano, Maurizio Elia, Rosaria Renna, Catello Vollono, Angelo Pascarella, Luciana Tramacere, Giovanni De Maria, Daniela Audenino, Maria Pia Pasolini, Loretta Giuliano, Rosita Galli, Gionata Strigaro, Monica Puligheddu, Angelo Labate, Pietro Penza, Stefano Quadri, David Stokelj, Giovanni Boero, Elisa Fallica, Monica Santo Sabato, Giovanni Falcicchio, and Nicoletta Foschi were responsible for patient enrollment and the collection of clinical data, and carried out data interpretation. Simona Lattanzi, Giancarlo Di Gennaro, Valentina Villano, Gabriele Camattari, Fabiano Mele, and Barbara Roncari revised the manuscript and provided substantial comments. The BLESS Study Group participated in the collection of study data (see Supplementary Materials).

## FUNDING INFORMATION

The BLESS study was funded by Angelini Pharma S.p.A.

## CONFLICT OF INTEREST STATEMENT

The authors declare the following financial interests/personal relationships, which may be considered as potential competing interests: Simona Lattanzi has received speaker or consultancy fees from Angelini Pharma, Eisai, GW Pharmaceuticals, Medscape, and UCB Pharma, and has served on advisory boards for Angelini Pharma, Arvelle Therapeutics, BIAL, Eisai, GW Pharmaceuticals, and Rapport Therapeutics. Fedele Dono has received speaker honoraria and travel grant from Eisai, Jazz Pharmaceuticals, and Angelini Pharma. Giuseppe d'Orsi has received speaker or consultancy fees from Angelini Pharma, Eisai, and UCB Pharma. Alfredo D'Aniello has received speaker honoraria from Angelini Pharma, UCB Pharma, and Eisai, and has participated in advisory board for Angelini Pharma. Mariangela Panebianco has received speaker or consultancy fees from Angelini Pharma, Eisai, and UCB Pharma. Paolo Bonanni has received speaker or consultancy fees from BIAL, Eisai, GW Pharmaceuticals, LivaNova, Lusofarmaco, Proveca, and Roche. Carlo Di Bonaventura has received consulting fees and honoraria from UCB Pharma, Eisai, GW Pharmaceuticals, BIAL, Angelini Pharma, Lusofarmaco, and Ecupharma. Antonio Gambardella has received speaker honoraria from Eisai, UCB Pharma, and Angelini Pharma. Federica Ranzato has received speaker fees from Angelini Pharma, Eisai, UCB Pharma, and LivaNova and has participated on an advisory board for Angelini Pharma. Giada Pauletto has received speaker's or consultancy fees from Eisai, Angelini Pharma, LivaNova, Lusofarmaco, and UCB Pharma. Elena Tartara has received speaker fees from Eisai and advisory board fees from Angelini Pharma. Angela La Neve has received speaker or consultancy fees from Eisai, Mylan, Sanofi, BIAL, GW Pharmaceuticals, UCB Pharma, Arvelle Therapeutics, Angelini Pharma, and Neuraxpharm. Francesca Bisulli has received consultancy fees from Angelini Pharma, UCB Pharma, Jazz Pharmaceuticals, and Eisai. Patrizia Pulitano, has received consulting fees or speaker honoraria from Angelini Pharma, UCB Pharma, and Eisai. Claudio Liguori has received research support and speaker honoraria from Angelini Pharma and has no other relevant conflict of interests related to this article. Vincenzo Belcastro has received consulting fees and honoraria from UCB Pharma, Angelini Pharma, Lusofarmaco, and Ecupharma. Chiara Pizzanelli has received consultancy fees from Angelini Pharma, Eisai, and UCB Pharma. Maurizio Elia has received speaker or consultancy fees from Angelini Pharma, Eisai, Lusofarmaco, Proveca, Takeda, and UCB Pharma. Rosaria Renna, has received speaker fees from Eisai. Daniela Audenino has received speaker or consultancy fees from Angelini Pharma and UCB Pharma. Loretta Giuliano has received speaker or consultancy fees from Eisai, Angelini Pharma, and Lusofarmaco. Rosita Galli has received speaker or consultancy fees from Angelini Pharma and Eisai. Gionata Strigaro has received fees from Eisai and Angelini Pharma. Monica Puligheddu has received speaker or consultancy fees from Angelini Pharma, Eisai, Lusofarmaco, Idorsia, Jazz Pharmaceuticals, Bioproject, Fidia, and UCB Pharma. Giovanni Boero has received speaker or consultancy fees from Eisai, Angelini Pharma, and UCB Pharma. Giovanni Falcicchio, has received speaker fees from Angelini Pharma. Michela Procaccini is an employee of Angelini Pharma, Italy. Valentina Villano is an employee of Angelini Pharma, Italy. Gabriele Camattari is an employee of Angelini Pharma, Italy. Fabiano Mele is an employee of IQVIA Solutions Italy SRL. Barbara Roncari is an employee of IQVIA Solutions Italy SRL. Giancarlo Di Gennaro has received speaker honoraria from Eisai, UCB Pharma, LivaNova, Lusofarmaco, and GW Pharmaceuticals; and has served on advisory boards for BIAL, Arvelle Therapeutics, Angelini Pharma, and UCB Pharma. The remaining authors have no conflicts of interest.

## ETHICS STATEMENT

All participants received a comprehensive explanation of the study procedures and goals, consistent with the Declaration of Helsinki (1964 and its later amendments), and voluntarily participated in this study after signing a written informed consent form. The study was approved by the ethics committees of all participating institutions before the start of data collection (first approval of the coordinating ethics committee – Comitato Etico IRCCS Istituto Neurologico Mediterraneo Neuromed – on September 29, 2022), and conducted under the guidelines for Good Pharmacoepidemiology Practices (GPP) and applicable regulatory requirements.^27^ We confirm that we have read the *Epilepsia* Journal's position on issues involved in ethical publication and affirm that this report is consistent with those guidelines.

## Supporting information


Table S1.

## Data Availability

The data that support the findings of this study are available from the corresponding author upon reasonable request.

## References

[1] World Health Organization . Epilepsy. 2024. https://www.who.int/en/news‐room/fact‐sheets/detail/epilepsy

[2] Sultana B , Panzini M‐A , Carpentier AV , Comtois J , Rioux B , Gore G , et al. Incidence and prevalence of drug‐resistant epilepsy: a systematic review and meta‐analysis. Neurology. 2021;96(17):805–817. 33722992 10.1212/WNL.0000000000011839

[3] Asadi‐Pooya AA , Brigo F , Lattanzi S , Blumcke I . Adult epilepsy. Lancet. 2023;402(10399):412–424. 37459868 10.1016/S0140-6736(23)01048-6

[4] van Hezik‐Wester V , de Groot S , Kanters T , Versteegh M , Wagner L , Ardesch J , et al. Burden of illness in people with medically refractory epilepsy who suffer from daily to weekly seizures: 12‐month follow‐up of participants in the EPISODE study. Front Neurol. 2022;13:1012486. 36388190 10.3389/fneur.2022.1012486 PMC9650114

[5] Connor GS , Williamson A . Effectiveness and safety of adjunctive cenobamate for focal seizures in adults with developmental disability treated in clinical practice. Epilepsy Behav Rep. 2022;18:100533. 35345772 10.1016/j.ebr.2022.100533 PMC8956884

[6] Villanueva V , Santos‐Carrasco D , Cabezudo‐Garcìa P , Gómez‐Ibáñez A , Garcés M , Serrano‐Castro P , et al. Real‐world safety and effectiveness of cenobamate in patients with focal onset seizures: outcomes from an expanded access program. Epilepsia Open. 2023;8:918–929. 37149853 10.1002/epi4.12757 PMC10472366

[7] Beltrán‐Corbellini A , Romeral‐Jimenéz M , Mayo P , Sánchez‐Miranda Román I , Iruzubieta P , Chico‐García JL , et al. Cenobamate in patients with highly refractory focal epilepsy: a retrospective real‐world study. Seizure: Eur J Epilepsy. 2023;111:71–77. 10.1016/j.seizure.2023.07.026 37549616

[8] Peña‐Ceballos J , Moloney P , Munteanu T , Naggar HE , Widdess‐Walsh P , Delanty N . Adjunctive cenobamate in highly active and ultra‐refractory focal epilepsy: a “real‐world” retrospective study. Epilepsia. 2023;126:24–31. 10.1111/epi.17549 36790345

[9] Roberti R , Assenza G , Bisulli F , Boero G , Canafoglia L , Chiesa V , et al. Adjunctive cenobamate in people with focal onset seizures: insights from the Italian expanded access program. Epilepsia. 2024;65:2909–2922. 39140704 10.1111/epi.18091

[10] Krauss GL , Klein P , Brandt C , Lee SK , Milanov I , Milovanovic M , et al. Safety and efficacy of adjunctive cenobamate (YKP3089) in patients with uncontrolled focal seizures: a multicentre, double‐blind, randomised, placebo‐controlled, dose‐response trial. Lancet Neurol. 2019;19:38–48. 31734103 10.1016/S1474-4422(19)30399-0

[11] Chung SS , French JA , Kowalski J , Krauss GL , Lee SK , Maciejowski M , et al. Randomised phase 2 study of adjunctive cenobamate in patients with uncontrolled focal seizures. Neurology. 2020;94:e2311‐22. 32409485 10.1212/WNL.0000000000009530 PMC7357293

[12] Lattanzi S , Trinka E , Zaccara G , Striano P , Del Giovane C , Silvestrini M , et al. Adjunctive Cenobamate for focal‐onset seizures in adults: a systematic review and meta‐analysis. CNS Drugs. 2020;34:1105–1120. 32851590 10.1007/s40263-020-00759-9 PMC7658084

[13] Klein P , Ferrari L , Rosenfeld WE . Cenobamate for the treatment of focal seizures. US Neurol. 2020;16(2):87–97.

[14] Sperling MR , Abou‐Khalil B , Aboumatar S , Bhatia P , Biton V , Klein P , et al. Efficacy of cenobamate for uncontrolled focal seizures: post hoc analysis of a phase 3, multicenter, open‐label study. Epilepsia. 2021;62(12):3005–3015. 34633084 10.1111/epi.17091 PMC9293007

[15] Rodríguez‐Uranga JJ , Sánchez‐Caro JM , Ramchandani RH . Treatment simplification to optimize cenobamate effectiveness and tolerability: a real‐world retrospective study in Spain. Epilepsia Open. 2024;9:1345–1356. 38800945 10.1002/epi4.12959 PMC11296129

[16] Winter Y , Abou Dargham R , Patiño Tobón S , Groppa S , Fuest S . Cenobamate as an early adjunctive treatment in drug‐resistant focal‐onset seizures: an observational cohort study. CNS Drugs. 2024;38(9):733–742. 10.1007/s40263-024-01109-9 39096467 PMC11316687

[17] Lattanzi S , Ranzato F , Di Bonaventura C , Bonanni P , Gambardella A , Tartara E , et al. Effectiveness and safety of adjunctive Cenobamate in people with focal‐onset epilepsy: evidence from the first interim analysis of the BLESS study. Neurol Ther. 2024;13(4):1203–1217. 10.1007/s40120-024-00634-5 38850402 PMC11263269

[18] Lattanzi S , Canafoglia L , Canevini MP , Casciato S , Chiesa V , Dainese F , et al. Adjunctive brivaracetam in focal epilepsy: real‐world evidence from the BRIVAracetam add‐on first Italian netwoRk study (BRIVAFIRST). CNS Drugs. 2021;35:1289–1301. 10.1007/s40263-021-00856-3 34476770 PMC8642333

[19] Schiller Y , Najjar Y . Quantifying the response to antiepileptic drugs. Effect of past treatment history. Neurology. 2008;70:54–65. 18166707 10.1212/01.wnl.0000286959.22040.6e

[20] Mecarelli O , Di Gennaro G , Vigevano F . Unmet needs and perspectives in management of drug resistant focal epilepsy: an Italian study. Epilepsy Behav. 2022;137:108950. 36347069 10.1016/j.yebeh.2022.108950

[21] Smith MC , Klei P , Krauss GL , Rashid S , Seiden LG , Stern JM , et al. Dose adjustment of concomitant antiseizure medications during Cenobamate treatment: expert opinion consensus recommendations. Neurol Ther. 2022;11:1705–1720. 36057761 10.1007/s40120-022-00400-5 PMC9588096

[22] Serrano‐Castro PJ , Ramírez‐García T , Cabezudo‐Garcia P , Garcia‐Martin G , de la Parra J . Effect of cenobamate on cognition in patients with drug‐resistant epilepsy with focal onset seizures: an exploratory study. CNS Drugs. 2024;38(2):141–151. 38265735 10.1007/s40263-024-01063-6 PMC10881647

[23] Steinhoff BJ , Georgiu D , Intravooth T . The cenobamate KORK study—a prospective monocenter observational study investigating cenobamate as an adjunctive therapy in refractory epilepsy, with comparisons to historical cohorts treated with add‐on lacosamide, perampanel, and brivaracetam. Epilepsia Open. 2024;9:1502–1514. 38861254 10.1002/epi4.12992 PMC11296107

[24] Aboumatar S , Ferrari L , Stern S , Wade CT , Weingarten M , Connor GS , et al. Reductions in concomitant antiseizure medication drug load during adjunctive cenobamate therapy: post‐hoc analysis of a subset of patients from a phase 3, multicenter, open‐label study. Epilepsy Res. 2024;200:107306. 38340681 10.1016/j.eplepsyres.2024.107306

[25] Friedo A , Greshake B , Makridis KL , Straub HB . Cenobamate significantly improves seizure control in intellectually disabled patients with drug‐resistant epilepsy and allows drug load reduction. Front Neurol. 2023;14:1209487. 37528853 10.3389/fneur.2023.1209487 PMC10390252

[26] Rosenfeld WE , Abou‐Khalil B , Aboumatar S , Bhatia P , Biton V , Krauss GL , et al. Post hoc analysis of a phase 3, multicenter, open‐label study of cenobamate for treatment of uncontrolled focal seizures: effects of dose adjustments of concomitant antiseizure medications. Epilepsia. 2021;62:3016–3028. 34633074 10.1111/epi.17092 PMC9292883

[27] Epstein M , on behalf of ISPE . Guidelines for good pharmacoepidemiology practices (GPP). Pharmacoepidemiol Drug Saf. 2016;25:2–10. 15918159 10.1002/pds.1082

