# Supplementary material for: Effectiveness and safety of adjunctive cenobamate in people with focal‐onset epilepsy: Interim results after 24‐week observational period from the BLESS study
Source: Epilepsia. 2025 Mar 15;66(7):2239–52. doi: 10.1111/epi.18357 (PMC12291020; doi:10.1111/epi.18357)
Supplement: Supplementary file 1 — Table S1. [file EPI-66-2239-s001.docx]

**SUPPLEMENTARY MATERIALS**

**Effectiveness and Safety of Adjunctive Cenobamate in People with Focal-Onset Epilepsy: Interim Results after 24-Week Observational Period from the BLESS Study**

**List of sites AND INVESTIGATORS participating on BLESS Study Group**

updated on June 6^th^, 2024 (date of database extraction for the Second Interim Statistical Analysis)

Alfredo D’Aniello (IRCCS Neuromed, Pozzilli - IS, Italy); Cecilia Catania, Alessandra Morano, Biagio Orlando, Maria Sole Borioni, Fabrizio Giaculli, Luca Pasquale Giordano (Policlinico Umberto I, Roma, Italy); Francesca Pia Mazzeo (Azienda Ospedaliero Universitaria Consorziale Policlinico, Bari, Italy); Marco Belluzzo (Azienda Sanitaria Universitaria Friuli Centrale-SOC Neurologia, Udine, Italy), Anna Carmen Nilo (Clinica Neurologica, Azienda Sanitaria Universitaria Friuli Centrale, Udine, Italy); Alessia Peretti, Diana Polo, Anna Bolzan (Ospedale San Bortolo, Vicenza, Italy); Giulia Lippa, Marianna Nesta, Lorenzo Ricci (Fondazione Policlinico Campus Bio-Medico, Roma, Italy); Alberto Danieli, Elisa Osanni (IRCCS E. Medea Scientific Institute, Epilepsy Unit, Conegliano, Treviso, Italy); Francesco Fortunato, Giuseppe Magro, Laura Marino, Ilaria Sammarra, Francesca Felicia Operto (Azienda Ospedaliero Universitaria “Renato Dulbecco”, Catanzaro, Italy); Carlo Andrea Galimberti, Domenico Lomonaco, Alessia Campomori (IRCCS Fondazione Mondino, Pavia, Università degli Studi di Pavia, Italy); Devis Collura, Emanuela Viglietta (Humanitas Gradenigo, Torino, Italy); Lorenzo Muccioli, Martina Soldà, Veronica Viola, Nicolò Locatelli (IRCCS Istituto delle Scienze Neurologiche di Bologna, full member ERN-EpiCARE, Bologna, Italy; Department of Biomedical and NeuroMotor Sciences - DIBINEM, University of Bologna, Bologna, Italy); Roberta Coa, Antonio Valentino Saporito (Azienda Ospedaliero-Universitaria Cagliari e Università di Cagliari, Monserrato - CA, Italy); Valeria Badioni, Maria Grazia Pascarella, Gionathan Mazza (Ospedale Maggiore, Lodi, Italy); Angelina Laganà, Rosario Beccore, Orazio Pardeo; Francesco Luppino, Serena Raineri (Neurophysiopathology and Movement Disorders Clinic, University of Messina, Messina, Italy); Tiziano Zanoni, Cecilia Zivelonghi (AOUI Verona Borgo Trento, Verona, Italy); Umberto Aguglia, Edoardo Ferlazzo, Vittoria Cianci, Roberta Cutellè (Department of medical and Surgical Sciences, Magna Graecia University of Catanzaro, Reggio Calabria, Italy); Laura Buttarelli, Roberto Matrullo, Davide Toraldo (Azienda Ospedaliero Universitaria Luigi Vanvitelli, Napoli, Italy); Chiara Di Blasi, Valeria Sarro, Antonio Volzone, Massimo Di Giovanni, Stefano Avventura, Maria Francesca Tepedino (Ambulatorio Epilessia, AOU San Giovanni di Dio e Ruggi d 'Aragona Clinica Neurologica, Salerno, Italy); Antonella Morea, Nicola Pilolli, Giuseppe Pontrelli (Ospedale SS. Annunziata, Taranto, Italy); Luigi Vetri (IRCCS Associazione Oasi Maria SS, Troina - EN, Italy); Sabina Bartalini, Giullia Maccanti, Giulia Beneduce, Damiano Bruno (Azienda Ospedaliero Universitaria Senese Policlinico Scotte, Siena, Italy); Stefano Luca Sensi, Giacomo Evangelista, Michelangelo Dasara, Clarissa Corniello, Stefano Consoli, Davide Liviello, Paolo Quintieri (CAST, Center for Advanced Studies and Technology ITAB, Institute for Advanced Biomedical Technologies, Department of Neuroscience, Imaging, and Clinical Science University “G. d’Annunzio” Chieti-Pescara, Chieti, Italy); Vincenzo Loreto (Azienda Ospedaliera di Rilievo Nazionale "A. Cardarelli" Ospedale Antonio Cardarelli, Napoli, Italy); Edward Cesnik, Carla Marcialis (Azienda Ospedaliero Universitaria Ferrara, Cona - FE, Italy); Martina Romozzi, Costanza Sottani (Fondazione Policlinico Universitario Agostino Gemelli, IRCCS, Roma, Italy); Matteo Antonucci (Department of Systems Medicine, University of Rome “Tor Vergata”, Roma, Italy); Alessio D’Elia, Laura Carmillo (Ospedale Vito Fazzi, Lecce, Italy); Lavinia Vassallo, Eloise Lo Mauro, Salvatore Maria Lima (Policlinico Giaccone, Palermo, Italy); Barbara Chiocchetti, Martina Calistri (Ospedale San Giovanni di Dio, Firenze, Italy); Andrea Scalvini, Elisabetta Del Zotto (Fondazione Poliambulanza, Brescia, Italy); Luigi Sicurella, Alice Figura, Giovanni Messina (ARNAS Garibaldi-UOC Neurologia, Catania, Italy); Marta Maria Tentorio, Paolo Costa, Chiara Silvestri, Elisabetta Rolla, Leandro Purin, Luca Colombo, Morgan Trevisan (ASST Spedali Civili, Brescia, Italy); Gabriele Mainini, Romano Giovanni Orofino (Fondazione IRCCS San Gerardo dei Tintori, University of Milano-Bicocca, Monza, Italy); Matteo Impellizzeri, Maria Cristina Servalli, Paolo Paone (ASST Papa Giovanni XXIII, Bergamo, Italy); Teresa Francavilla, Daniele Graziani (Neurologia Universitaria "Puca-Amaducci", Azienda Ospedaliero Universitaria Consorziale Policlinico, Bari, Italy); Pamela Rosettani, Sara Zagaglia, Luciana Mollo, Azzurra Biagiotti, Alessandro Alesi (Azienda Ospedaliero Universitaria delle Marche, Ancona, Italy); Maria Teresa Di Claudio (IRCCS Ospedale Casa Sollievo della Sofferenza-UOC Neurologia, San Giovanni Rotondo - FG, Italy); Pierangela Riani, Fabio Iannaccone, Claudia Scarpitta (Neurology Unit, Department of Clinical and Experimental Medicine, University of Pisa, Pisa, Italy); Maria Proietto, Angelo Battiato, Salvatore Dominici (UOC Clinica Neurologica, AOU Policlinico G. Rodolico San Marco, Catania, Italy); Martina Guadagni, Daniela Marino (Ospedale San Donato, Arezzo, Italy); Rossella Papetti, Marzia Carlini, Lucia Farotti (Ospedale Santa Maria della Misericordia, Perugia, Italy); Marinella Tomaselli, Stefania Pavan, Matteo Cegalin (Azienda Sanitaria Universitaria Giuliano Isontina - ASUGI, Trieste, Italy); Alessandra Di Liberto, Giovanni Battista Rossi, Edoardo Della Sala, Claudio Gojani, Andrea Novara, Camilla Bertolini (Città della Salute e Scienza PO Molinette, Torino, Italy); Edoardo Pronello, Francesca Lozza (Azienda Ospedaliero Universitaria Maggiore della Carità, Novara, Italy).

**Figure S1. BLESS Study subject disposition of interim analysis**


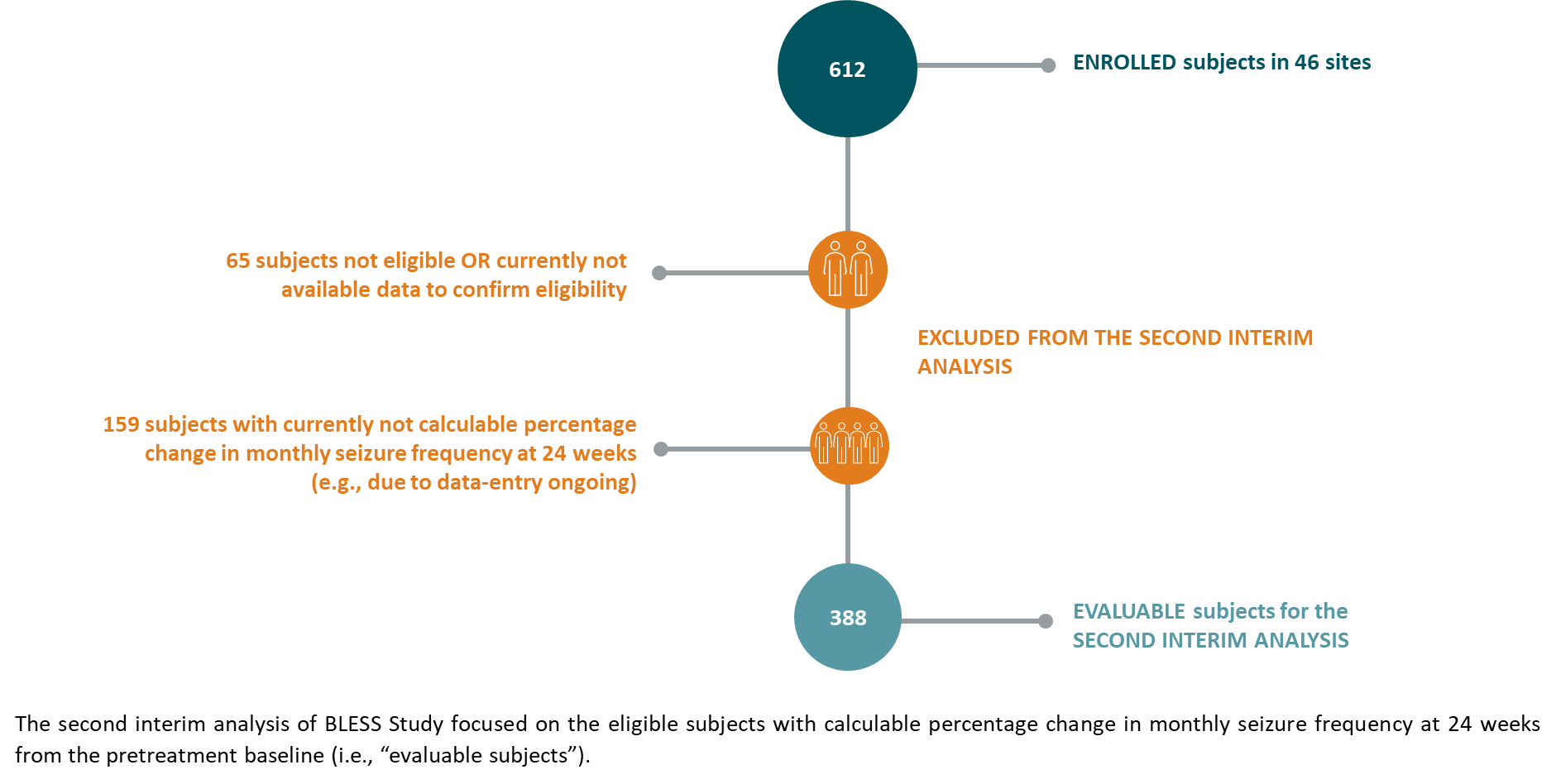


**Table S1.** **Ongoing medical conditions and treatment history at baseline**

|  | All  (N=388) | 2-3 previous ASMs (N=76) | >3 previous ASMs  (N=312) |
| --- | --- | --- | --- |
| **Ongoing medical conditions/comorbidities at cenobamate**  **treatment initiation**^a,b^, n (%) | | | |
| Developmental disorder  Anxiety  Hypertension  Thyroid condition  Depression  Dyslipidemia  Brain neoplasia (not malignant)  Migraine  Allergies  Brain vascular malformation  Cognitive impairment  Diabetes mellitus  Hearing loss  Psychosis  Heart failure  Insomnia  Obstructive sleep apnea  Arthritis  Hepatic impairment  Asthma  Atrial fibrillation  Malignancy-solid organ  Myocardial infarction  Peripheral vascular disease  Cataracts  Glaucoma  Pulmonary disease  Renal impairment  Other ongoing condition/comorbidities | 50 (12.9)  27 (7.0)  26 (6.7)  26 (6.7)  25 (6.4)  23 (5.9)  12 (3.1)  12 (3.1)  9 (2.3)  9 (2.3)  8 (2.1)  6 (1.5)  6 (1.5)  6 (1.5)  5 (1.3)  5 (1.3)  5 (1.3)  4 (1.0)  3 (0.8)  2 (0.5)  2 (0.5)  2 (0.5)  2 (0.5)  2 (0.5)  1 (0.3)  1 (0.3)  1 (0.3)  1 (0.3)  99 (25.5) | 7 (9.2)  3 (3.9)  8 (10.5)  8 (10.5)  6 (7.9)  5 (6.6)  2 (2.6)  4 (5.3)  0 (0.0)  1 (1.3)  0 (0.0)  4 (5.3)  0 (0.0)  2 (2.6)  2 (2.6)  1 (1.3)  2 (2.6)  1 (1.3)  0 (0.0)  0 ( 0.0)  1 (1.3)  0 (0.0)  0 (0.0)  1 (1.3)  0 (0.0)  0 (0.0)  0 (0.0)  0 (0.0)  9 (11.8) | 43 (13.8)  24 (7.7)  18 (5.8)  18 (5.8)  19 ( 6.1)  18 (5.8)  10 (3.2)  8 (2.6)  9 ( 2.9)  8 (2.6)  8 (2.6)  2 (0.6)  6 (1.9)  4 (1.3)  3 (1.0)  4 (1.3)  3 (1.0)  3 (1.0)  3 (1.0)  2 (0.6)  1 (0.3)  2 (0.6)  2 (0.6)  1 (0.3)  1 (0.3)  1 (0.3)  1 (0.3)  1 (0.3)  90 (28.8) |

^a^ Subjects could have more than one condition of interest.

^b^ Percentages were computed excluding subjects with missing data from the total.

ASM: anti-seizures medication; IQR: interquartile range.

The reported results refer to the second interim analysis of BLESS Study.

**Table S2. Participants per any concomitant anti-seizure medications at start of treatment and at 12 weeks and 24 weeks after treatment initiation**

|  | All  (N=388) | | | 2-3 previous ASMs  (N=76) | | | >3 previous ASMs  (N=312) | | |
| --- | --- | --- | --- | --- | --- | --- | --- | --- | --- |
|  | **Index date** | **12 weeks** | **24 weeks** | **Index date** | **12 weeks** | **24 weeks** | **Index date** | **12 weeks** | **24 weeks** |
| Participants per any concomitant ASMs, n (%)^a,b^ |  |  |  |  |  |  |  |  |  |
| Lacosamide | 121 (31.5) | 117 (30.4) | 107 (27.8) | 27 (35.5) | 27 (35.5) | 24 (31.6) | 94 (30.5) | 90 (29.1) | 83 (26.9) |
| Carbamazepine | 110 (28.6) | 105 (27.3) | 101 (26.2) | 17 (22.4) | 17 (22.4) | 17 (22.4) | 93 (30.2) | 88 (28.5) | 84 (27.2) |
| Brivaracetam | 107 (27.9) | 102 (26.5) | 100 (26.0) | 11 (14.5) | 11 (14.5) | 11 (14.5) | 96 (31.2) | 91 (29.4) | 89 (28.8) |
| Perampanel | 89 (23.2) | 83 (21.6) | 82 (21.3) | 9 (11.8) | 8 (10.5) | 8 (10.5) | 80 (26.0) | 75 (24.3) | 74 (23.9) |
| Lamotrigine | 76 (19.8) | 76 (19.7) | 75 (19.5) | 13 (17.1) | 13 (17.1) | 13 (17.1) | 63 (20.5) | 63 (20.4) | 62 (20.1) |
| Levetiracetam | 72 (18.8) | 70 (18.2) | 66 (17.1) | 26 (34.2) | 25 (32.9) | 22 (28.9) | 46 (14.9) | 45 (14.6) | 44 (14.2) |
| Valproic Acid | 62 (16.1) | 61 (15.8) | 60 (15.6) | 5 (6.6) | 5 (6.6) | 5 (6.6) | 57 (18.5) | 56 (18.1) | 55 (17.8) |
| Clobazam | 58 (15.1) | 53 (13.8) | 52 (13.5) | 5 (6.6) | 5 (6.6) | 5 (6.6) | 53 (17.2) | 48 (15.5) | 47 (15.2) |
| Topiramate | 43 (11.2) | 40 (10.4) | 39 (10.1) | 2 (2.6) | 2 (2.6) | 2 (2.6) | 41 (13.3) | 38 (12.3) | 37 (12.0) |
| Phenobarbital | 34 (8.9) | 32 (8.3) | 32 (8.3) | 3 (3.9) | 3 (3.9) | 3 (3.9) | 31 (10.1) | 29 (9.4) | 29 (9.4) |
| Oxcarbazepine | 33 (8.6) | 31 (8.1) | 30 (7.8) | 4 (5.3) | 4 (5.3) | 4 (5.3) | 29 (9.4) | 27 (8.7) | 26 (8.4) |
| Clonazepam | 31 (8.1) | 27 (7.0) | 28 (7.3) | 3 (3.9) | 3 (3.9) | 3 (3.9) | 28 (9.1) | 24 (7.8) | 25 (8.1) |
| Zonisamide | 27 (7.0) | 27 (7.0) | 22 (5.7) | 1 (1.3) | 1 (1.3) | 1 (1.3) | 26 (8.4) | 26 (8.4) | 21 (6.8) |
| Phenytoin | 15 (3.9) | 13 (3.4) | 11 (2.9) | 2 (2.6) | 2 (2.6) | 2 (2.6) | 13 (4.2) | 11 (3.6) | 9 (2.9) |
| Divalproex Sodium | 4 (1.0) | 4 (1.0) | 4 (1.0) | 0 (0.0) | 0 (0.0) | 0 (0.0) | 4 (1.3) | 4 (1.3) | 4 (1.3) |
| Ethosuximide | 1 (0.3) | 1 (0.3) | 1 (0.3) | 0 (0.0) | 0 (0.0) | 0 (0.0) | 1 (0.3) | 1 (0.3) | 1 (0.3) |
| Gabapentin | 1 (0.3) | 1 (0.3) | 1 (0.3) | 0 (0.0) | 0 (0.0) | 0 (0.0) | 1 (0.3) | 1 (0.3) | 1 (0.3) |
| Tiagabine HCl | 1 (0.3) | 1 (0.3) | 1 (0.3) | 0 (0.0) | 0 (0.0) | 0 (0.0) | 1 (0.3) | 1 (0.3) | 1 (0.3) |
| Other Medication | 41.0 (10.7) | 35 (9.1) | 34 (8.8) | 5 (6.6) | 5 (6.6) | 5 (6.6) | 36 (11.7) | 30 (9.7) | 29 (9.4) |
| Missing | 4 | 3 | 3 | 0 | 0 | 0 | 4 | 3 | 3 |

^a^ Percentages were computed excluding subjects with missing data from the total.

^b^ Participants could have taken more than one concomitant ASM (cenobamate was not counted as concomitant therapy).

Index date: date of initiation of cenobamate treatment.

ASM: anti-seizure medication.

The reported results refer to the second interim analysis of the BLESS Study.

**Table S3. Summary of ADRs to cenobamate until 24 weeks after cenobamate treatment initiation**

|  | **All**  **(N=388)** | **2-3 previous** **ASMs**  **(N=76)** | **>3 previous ASMs**  **(N=312)** |
| --- | --- | --- | --- |
| Subjects experiencing ADRs to cenobamate until 24 weeks after cenobamate treatment initiation, n (%) | 77 (19.8) | 4 (5.3) | 73 (23.4) |
| **Nervous system disorders** | **58 (14.9)** | **4 (5.3)** | **54 (17.3)** |
| Ataxia | 5 (1.3) | 0 (0.0) | 5 (1.6) |
| Balance disorder | 8 (2.1) | 0 (0.0) | 8 (2.6) |
| Change in seizure presentation | 2 (0.5) | 0 (0.0) | 2 (0.6) |
| Disturbance in attention | 2 (0.5) | 0 (0.0) | 2 (0.6) |
| Dizziness | 10 (2.6) | 1 (1.3) | 9 (2.9) |
| Dysarthria | 5 (1.3) | 0 (0.0) | 5 (1.6) |
| Headache | 2 (0.5) | 1 (1.3) | 1 (0.3) |
| Memory impairment | 3 (0.8) | 0 (0.0) | 3 (1.0) |
| Somnolence | 38 (9.8) | 3 (3.9) | 35 (11.2) |
| **Eye disorders** | **10 (2.6)** | **0 (0.0)** | **10 (3.2)** |
| Diplopia | 7 (1.8) | 0 (0.0) | 7 (2.2) |
| Visual impairment | 2 (0.5) | 0 (0.0) | 2 (0.6) |
| Xerophthalmia | 1 (0.3) | 0 (0.0) | 1 (0.3) |
| **Psychiatric disorders** | **8 (2.1)** | **0 (0.0)** | **8 (2.6)** |
| Anxiety | 1 (0.3) | 0 (0.0) | 1 (0.3) |
| Confusional state | 1 (0.3) | 0 (0.0) | 1 (0.3) |
| Depression | 3 (0.8) | 0 (0.0) | 3 (1.0) |
| Psychomotor retardation | 4 (1.0) | 0 (0.0) | 4 (1.3) |
| **General disorders and administration site conditions** | **5 (1.3)** | **0 (0.0)** | **5 (1.6)** |
| Asthenia | 2 (0.5) | 0 (0.0) | 2 (0.6) |
| Exercise tolerance decreased | 1 (0.3) | 0 (0.0) | 1 (0.3) |
| Fatigue | 1 (0.3) | 0 (0.0) | 1 (0.3) |
| Generalised oedema | 1 (0.3) | 0 (0.0) | 1 (0.3) |
| **Ear and labyrinth disorders** | **4 (1.0)** | **0 (0.0)** | **4 (1.3)** |
| Vertigo | 4 (1.0) | 0 (0.0) | 4 (1.3) |
| **Blood and lymphatic system disorders** | **2 (0.5)** | **0 (0.0)** | **2 (0.6)** |
| Anaemia | 1 (0.3) | 0 (0.0) | 1 (0.3) |
| Leukopenia | 2 (0.5) | 0 (0.0) | 2 (0.6) |
| Thrombocytopenia | 1 (0.3) | 0 (0.0) | 1 (0.3) |
| **Skin and subcutaneous tissue disorders** | **2 (0.5)** | **0 (0.0)** | **2 (0.6)** |
| Erythema | 1 (0.3) | 0 (0.0) | 1 (0.3) |
| Rash | 1 (0.3) | 0 (0.0) | 1 (0.3) |
| **Gastrointestinal disorders** | **1 (0.3)** | **0 (0.0)** | **1 (0.3)** |
| Nausea | 1 (0.3) | 0 (0.0) | 1 (0.3) |
| **Injury, poisoning and procedural complications** | **1 (0.3)** | **0 (0.0)** | **1 (0.3)** |
| Craniofacial injury | 1 (0.3) | 0 (0.0) | 1 (0.3) |
| **Investigations** | **1 (0.3)** | **0 (0.0)** | **1 (0.3)** |
| Gamma-glutamyltransferase increased | 1 (0.3) | 0 (0.0) | 1 (0.3) |
| **Uncoded** | **3 (0.8)** | **0 (0.0)** | **3 (1.0)** |

^a^A patient could have more than one ADR occurring until 24 weeks after cenobamate treatment initiation. Recoding based on the Medical Dictionary for Regulatory Activities (MedDRA®) Version 26.0 March 2023. MedDRA® trademark is registered by the International Council for Harmonisation of Technical Requirements for Pharmaceuticals for Human Use (ICH).

ADR: Adverse Drug Reaction; ASM: anti-seizure medication.

The reported results refer to the second interim analysis of the BLESS Study.
